# The Development of Joint Kinematics, Kinetics and Electromyography Activity over 50 m Sprints in Experienced Sprinters

**DOI:** 10.3390/jfmk11030352

**Published:** 2026-09-03

**Authors:** Roland van den Tillaar, Sam Gleadhill, Pedro Jiménez-Reyes, Ryu Nagahara

**Affiliations:** 1 Department of Sports Sciences, Nord University, 7600 Levanger, Norway; 2 Alliance for Research in Exercise, Nutrition and Activity (ARENA), Allied Health and Human Performance, University of South Australia, Adelaide, SA 5000, Australia; sam.gleadhill@adelaide.edu.au; 3 Centre for Sport Studies, Rey Juan Carlos University, 28943 Madrid, Spain; pedro.jimenezr@urjc.es; 4 National Institute of Fitness and Sports in Kanoya, Kanoya 891-2393, Japan; nagahara@nifs-k.ac.jp

**Keywords:** angles, segmental angular velocity, ground reaction forces

## Abstract

**Background:** The aim was to investigate the stride-by-stride development of spatiotemporal parameters, joint kinematics, kinetics, and electromyographic (EMG) activity during a 50 m sprint, providing a comprehensive analysis of the biomechanical and neuromuscular changes throughout the entire sprint. **Methods:** Fifteen male sprinters (age 20.5 ± 1.3 years, body mass 66.8 ± 4.5 kg, body height 1.74 ± 0.06 m, 100 m personal best: 11.24 ± 0.34 s) performed a 50 m sprint, while the spatiotemporal parameters, joint kinematics, kinetics, and EMG activity of nine muscles were measured. **Results:** The main findings were that the 50 m sprint could be divided into three phases: initial acceleration, transition and maximal velocity and involve tightly coordinated changes across multiple biomechanical domains. The initial acceleration (the first 3–5 strides) was dominated by rapid increases in stride frequency, rapid changes in joint angles, and rising EMG activity in key muscles. In the transition phase (up to ~30–35 m), continued increases in stride length, decreasing contact time, and stabilization of kinematics and EMG activity were the main characteristics. In the maximal-velocity phase, a plateau in velocity, increased flight time, greater reliance on vertical force (an increase in vertical force relative to the horizontal forces), and stabilized neuromuscular patterns were observed. **Conclusion:** The phased progression supports contemporary biomechanical models of sprinting and emphasizes the importance of both mechanical and neuromuscular efficiency in achieving optimal sprint performance.

## 1. Introduction

Sprinting is a fundamental determinant of success in many sports, including athletics and field-based sports such as football and rugby, where the ability to accelerate rapidly and achieve high running velocities often determines competitive outcomes. In sprint events, overall performance is largely determined by an athlete’s ability to generate high acceleration and achieve maximal velocity within a limited distance [1,2]. Consequently, understanding the biomechanical and neuromuscular mechanisms underlying sprint performance has been a major focus of research within sports biomechanics and physiology for several decades.

Sprint velocity is determined by the product of stride length and stride frequency, which together define the spatiotemporal characteristics of sprinting [3]. During sprint acceleration, athletes progressively increase velocity through stride-by-stride adjustments in these variables, typically characterized by increasing stride length and decreasing ground contact times as speed increases [4,5]. Early acceleration is generally associated with longer ground contact times, larger propulsive impulses, and a pronounced forward body lean that facilitates horizontal force production [6,7]. As athletes transition toward maximal velocity, stride frequency increases and body posture gradually becomes more upright, reflecting important mechanical and technical adaptations required to sustain high running speeds [4,8]. These spatiotemporal adjustments illustrate the highly dynamic nature of sprinting, where stride characteristics evolve continuously throughout the sprint [9].

Stride length and frequency are performance outcome variables and are influenced by other kinematic and kinetic factors, including joint angles, segment velocities, and ground reaction forces. The ability to apply greater ground reaction forces in a shorter time generally results in faster sprinting [10], where the orientation of these forces relative to the ground is a key determinant of sprint acceleration performance. In particular, the capacity to effectively orient ground reaction forces in the horizontal direction has been identified as a distinguishing characteristic of faster sprinters [11]. For the generation and transmission of these forces, joint kinematics play a critical role during sprinting. Coordinated movements of the hip, knee, and ankle joints allow athletes to optimize propulsion while minimizing braking forces during ground contact [12]. For example, hip extension and plantar flexion contribute substantially to propulsive force generation, while knee flexion during the swing phase facilitates rapid limb repositioning and preparation for ground contact [13]. Additionally, the progressive transition from a forward-leaning posture during early acceleration toward a more upright posture as athletes approach maximal velocity reflects important adjustments in lower-limb kinematics and whole-body mechanics [4,7]. These findings highlight the complex interactions between the kinematic and kinetic variables that underpin sprint performance.

To develop and change biomechanical variables in sprint running, neuromuscular activation patterns play an essential role. Electromyography (EMG) studies have shown that muscles such as the gluteus maximus, hamstrings, quadriceps, and plantar flexors exhibit phase-specific activation patterns that contribute to propulsion, limb repositioning, and stabilization of the lower extremities during sprinting [14,15]. The hamstring muscles, in particular, play a critical role during the late swing phase by decelerating the swinging limb and preparing the leg for ground contact while also contributing to hip extension during the stance phase [13,16]. These neuromuscular strategies interact closely with biomechanical variables to enable the production of large forces within the short ground contact times characteristic of sprinting.

Despite the extensive body of research investigating sprint biomechanics, most studies have examined individual components of sprint performance in isolation, such as spatiotemporal parameters, ground reaction forces, joint kinematics, or muscle activation patterns. Furthermore, many investigations have focused on limited sections of the sprint, such as the first few strides of acceleration or the maximal-velocity phase [3,8,17]. While these studies have provided valuable insights into the determinants of sprint performance, they do not fully capture the complex interactions between biomechanical and neuromuscular variables that occur throughout the entire sprint acceleration until the maximal velocity.

Comprehensive, stride-by-stride analyses that simultaneously integrate spatiotemporal parameters, joint kinematics, kinetics, and neuromuscular activation patterns across the full acceleration and maximal-velocity phases remain limited in the literature. Most previous studies have typically examined these variables in isolation or focused on specific sections of the sprint, which may overlook the dynamic interactions that occur across successive strides.

Furthermore, recent approaches have emphasized the importance of analyzing sprint performance from a phase-specific and mechanically oriented perspective, highlighting how distinct mechanical demands emerge across the acceleration and maximal-velocity phases [18]. However, an integrated description combining mechanical and neuromuscular variables across these phases is still lacking. Given that sprinting is a highly dynamic movement in which mechanical and neuromuscular variables evolve continuously across successive strides, such an integrated approach may provide a more complete understanding of the mechanisms underpinning sprint performance and the strategies used by experienced sprinters to generate and sustain high running velocities. Therefore, the aim of this study was to investigate the stride-by-stride development of spatiotemporal parameters, joint kinematics, kinetics, and EMG activity during a 50 m sprint in experienced sprinters, providing a comprehensive analysis of the biomechanical and neuromuscular changes occurring throughout both the acceleration and maximal-velocity phases of sprinting. It was hypothesized that different parts of the 50 m sprint demand different joint kinematics, kinetics and EMG activities of the muscles and that these different analyses are related to each other. Information from all these parameters can give a better understanding of the different strides from initial acceleration to maximal velocity during sprinting, which could help sprinters and coaches optimize training strategies.

## 2. Methods

### 2.1. Participants

Fifteen experienced male sprinters (age 20.5 ± 1.3 years, body mass 66.8 ± 4.5 kg, body height 1.74 ± 0.06 m) with a personal-best 100 m time of 11.24 ± 0.34 s were recruited from the local university athletics club where they primarily trained in sprinting and/or horizontal jumps. They had to be in good health and free from injuries or sickness that could influence their sprint performance to be included in the study. Informed consent was obtained from each participant prior to the study. The study complied with current ethical regulations for research and was approved by the institutional research ethics committee.

### 2.2. Testing Procedure

**Figure 1:**
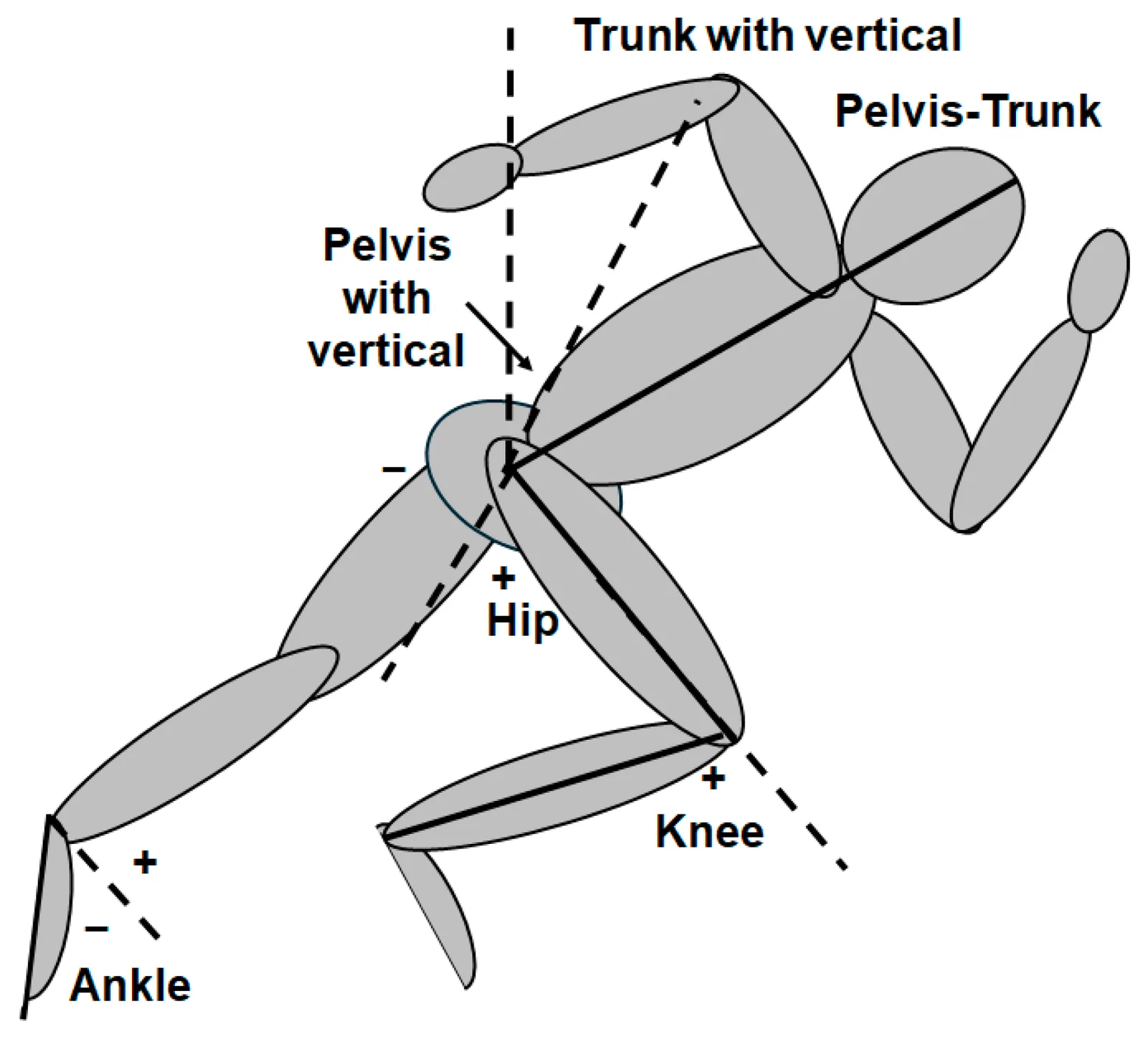
Definition of joint angles of trunk, pelvis, hip, knee and ankle around sagittal plane.

The athletes completed three sessions: two familiarization sessions to become familiar with the experimental setup, as the testing was part of a larger study, and one testing session, with a minimum of two days separating each. The testing session took place on an indoor athletic track. The primary data-collection session was conducted on a 110 m indoor track equipped with a 52 m force platform system comprising 54 units (models TF-90100, TF-3055, TF-32120; Tec Gihan, Uji, Japan) operating at 1000 Hz [18]. After a self-selected warm-up (in general consisting of jogging, dynamic exercise to increase range of motion and some short sprints at submaximal level, but different in length for each subject to have them fully prepared), participants performed one to two unresisted 50 m maximal sprints from a three-point start while wearing spikes. Before the test session, each participant put on an Xsens Lycra suit (Xsens Technologies B.V., Enschede, The Netherlands) containing 17 inertial measurement units (IMUs; 240 Hz) attached to the spikes, shanks, thighs, pelvis, sternum, head, upper arms, and forearms. Touchdown and toe-off for each stride with the right foot were identified using the algorithm described by van den Tillaar, et al. [19] and subsequently verified through visual inspection and software analysis (MVN Analyze 2025.4, Xsens, The Netherlands). Only strides with the right foot were analyzed as only on that side was EMG activity measured to avoid an excessive number of comparisons if evaluated step-by-step. Joint angles in the sagittal plane (ankle, knee, hip, pelvis, and trunk) were extracted for further analysis. In addition, the minimal and maximal joint angles (ankle, knee, and hip; see Figure 1) and maximal angular velocities of these joints in the sagittal plane during the contact and flight phases of each stride with the right foot were recorded (MVN Analyze 2025.4, Xsens, The Netherlands).

Distance-over-time measurements were recorded continuously during the 50 m sprints using a CMP3 Distance Sensor laser gun (Noptel Oy, Oulu, Finland), sampling at 2.56 kHz [20]. Velocity was automatically calculated as part of the Musclelab system (Ergotest Technology AS, Statthelle, Norway). Two wireless, 9-degree-of-freedom IMUs integrated with a 3-axis gyroscope (sampling rate of 1000 Hz) were attached on top of the shoelaces of the spikes of each foot to identify contact and flight times automatically using the software, together with stride length (defined as the distance between two consecutive contacts of the right foot measured with a laser) and frequency (1/contact + flight time stride) as the laser was synchronized with the Musclelab software v. 10.251 (Ergotest Technology AS, Langesund, Norway), which had very high correlations (r > 0.92) with a 50 m force plate system [19]. The contact and flight times of the right leg were used for further analysis as the EMG sensors were attached to the right leg, for which EMG activity was averaged for a flight and contact phase [21].

Captured kinetic data was filtered through a digital 50 Hz low-pass, fourth-order Butterworth filter. Ground reaction forces at every stride were calculated in reference to previous studies [22,23]. For each stride with the right foot, the contact phase, braking, propulsion and mean net anteroposterior together with vertical forces (N) were assessed. Furthermore, the ratio of force was calculated for each stride-by-stride-averaged mean net anterior force divided by the stride-averaged vertical force [24]. Additionally, the net anteroposterior and vertical impulses (N·s) during the contact phase were computed by integrating the ground reaction forces in the respective directions. Finally, the effective vertical mean force and impulse during the contact phase were computed by subtracting the body mass from the vertical force and then integrating it with respect to duration [10,25]. All ground reaction force (N/kg) and impulse variables were divided by body mass (N·s/kg).

The EMG activity of the muscles was measured by using a wireless unit with a sampling rate of 1 kHz (Ergotest Innovation, Porsgrunn, Norway) with Dri-Stick Silver circular sEMG AE-131 (NeuroDyne Medical, Germantown, TN, USA) electrodes on the muscles of the right lower limb [21] as we expected that there would be no mean differences in EMG activity between the left and right legs during sprints. The skin to which the electrodes were fastened had been shaved and washed with alcohol before fastening the electrodes. The electrodes (11 mm contact diameter and 2 cm centre-to-centre distance) were placed along the presumed direction of the underlying muscle fibres on the lateral and medial vastii, rectus femoris, biceps femoris, semimembranosus, soleus, lateral gastrocnemius, gluteus medius, and gluteus maximus muscles according to the recommendations of SENIAM [26]. To keep the electrodes in their place during sprinting, they were taped over, and the Lycra suit also secured their placement on the skin. The EMG raw signal was amplified by 400 and filtered using a preamplifier located as close as possible to the pickup point with the intention of minimizing the noise induced from external sources through the signal cables. The preamplifier had a common-mode rejection ratio of 100 dB. The raw EMG signals were band-pass (fourth-order Butterworth filter) filtered with a cutoff frequency of 20 Hz and 500 Hz, rectified, integrated and converted to root-mean-square (RMS) signals using a hardware circuit network (frequency response 450 kHz, averaging constant 12 ms, total error ± 0.5%) [27] for the contact and flight phases of each stride on right side. All sensors were synchronized using Musclelab version 10.251 (Ergotest Innovation, Porsgrunn, Norway), which made it possible to measure and analyze the kinematics and muscle activity for each stride cycle [21]. To compare EMG activity between the strides, EMG normalization was performed by using the highest mean RMS of a phase (contact or flight) during one stride of the 50 m sprint for each muscle as a normalization signal for each participant. This has been shown to be a reliable, repeatable and sensitive method for normalization of EMG activity in sprinting [28,29]. All systems (Musclelab, Xsens and force plates) were synchronized by the IMU sensors attached on top of the spikes fixed by tape exactly under the IMUs of the Xsens system (Xsens and Musclelab), which allowed these systems to identify touchdown and toe-off events (abrupt dip in angular velocity and increase in acceleration of IMU [30] synchronically, as both IMUs showed the same signals which could recognize these events. These events and phases were also clearly identifiable with the force plate system, which made it possible to synchronize the signals for all three systems as described by van den Tillaar, et al. [19] in an earlier study.

### 2.3. Statistics

To investigate the development over the different strides, spatiotemporal parameters, joint kinematics, kinetics and EMG activity over 50 m, a one-way ANOVA with repeated measurements was performed. To compare EMG activity in the flight and contact phases, a 2 (phase: contact, flight) × 13 (stride) ANOVA with repeated measures was performed. When significant differences were observed, post hoc comparisons with Holm–Bonferroni correction were performed. Effect size was evaluated with eta squared (η^2^) where 0.01 < η^2^ < 0.06 constitutes a small effect, 0.06 < η^2^ < 0.14 a medium one and η^2^ > 0.14 a large effect [31]. Where the sphericity assumption was violated, the Greenhouse–Geisser adjustments of the *p*-values were reported. The level of significance was set at *p* < 0.05. All data analyses were performed using JASP v. 0.19.1 (University of Amsterdam, Amsterdam, The Netherlands). Data are presented as means and 95% confidence intervals.

## 3. Results

### 3.1. Spatiotemporal Parameters

**Figure 2:**
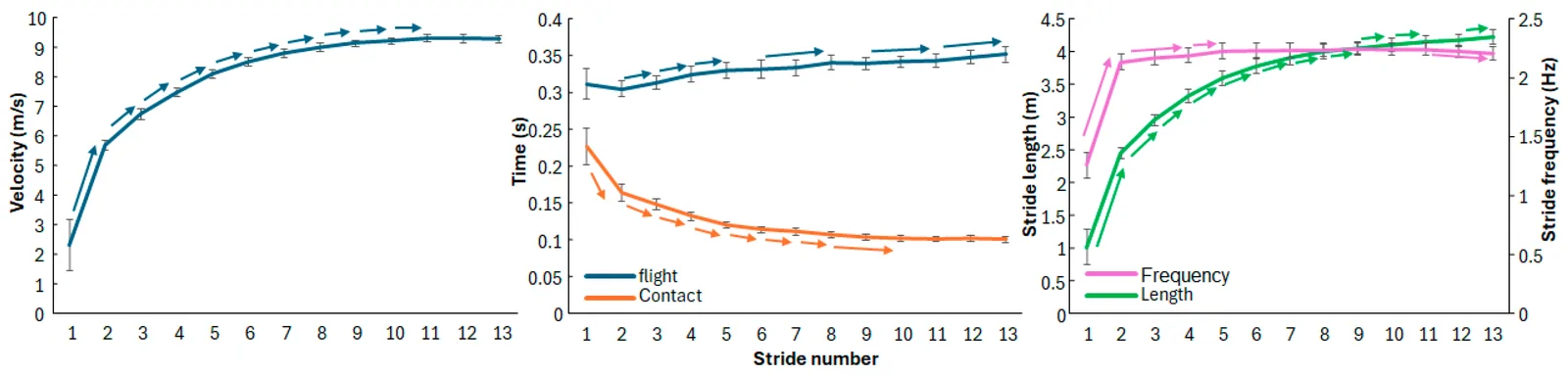
Mean ± 95% CI velocity and spatiotemporal parameters per stride. → indicates a significant change between these two strides and all strides right from the arrow.

Maximal velocity and all other spatiotemporal parameters were significantly affected across the strides (F ≥ 33.2, *p* < 0.001, η^2^ ≥ 0.68). Post hoc comparison revealed that stride length increased significantly with each stride, except between strides 11 and 12, while velocity increased every stride until stride eleven, which corresponded to approximately 35.1 ± 1.7 m. Stride frequency increased significantly during the first five strides and decreased between strides 11 and 13. In addition, contact time decreased significantly with each stride until stride 8 and then again between 8 and 10, while flight times increased each stride from stride 2 to 5 and then again between 6 and 8, 9 and 11 and 11 and 13 (Figure 2).

### 3.2. Joint Kinematics

**Figure 3:**
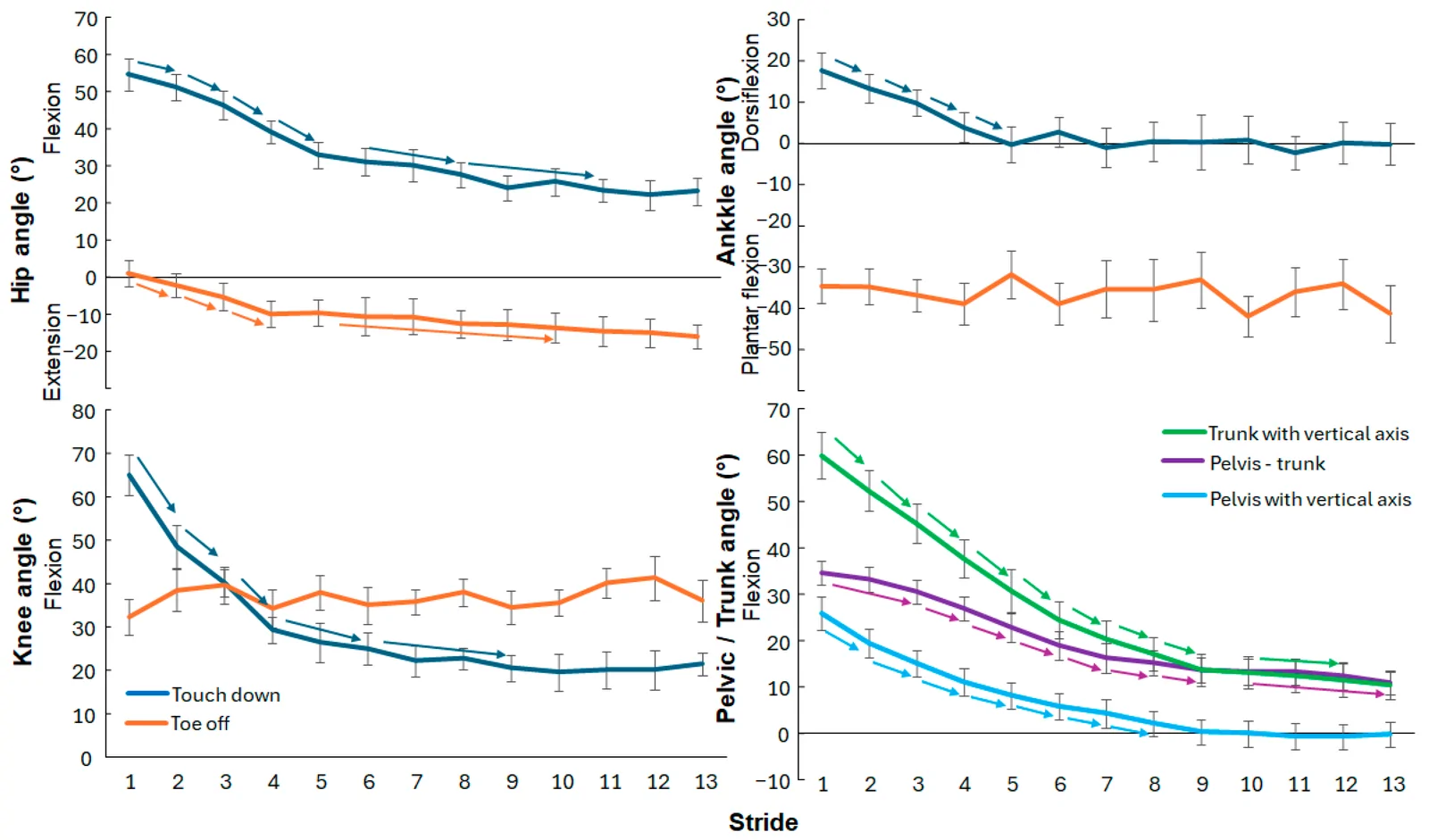
Mean ± 95% CI joint angles at touchdown and toe-off for each stride. → indicates a significant change between these two strides and all subsequent data points located to the right of the arrow.

**Figure 4:**
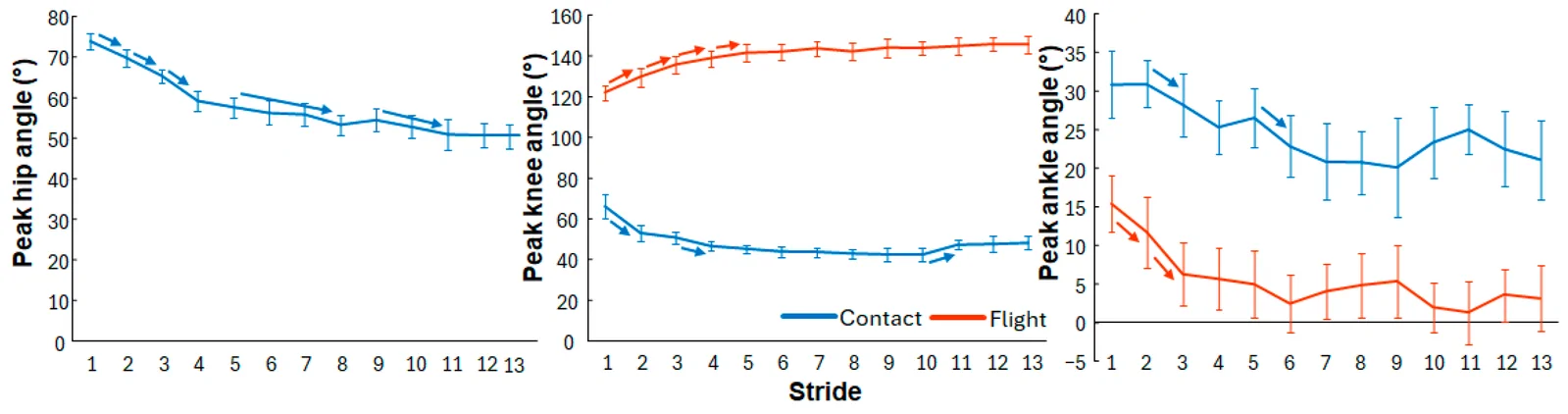
Mean ± 95% CI peak hip flexion and knee flexion during the flight phase and peak dorsiflexion during the contact phase for each stride. → indicates a significant change between these two strides and all subsequent data points located to the right of the arrow.

All the joint angles at touchdown, toe-off and maximal angles were significantly affected by the strides (F ≥ 8.5, *p* < 0.01, η^2^ ≥ 0.40) except for knee flexion and plantar flexion at toe-off (F = 1.78, *p* = 0.06, η^2^ = 0.12). Post hoc comparison revealed that touchdown hip, knee flexion and ankle dorsiflexion angles decreased each stride during the first 4–5 strides and thereafter decreased less and stabilized, while at toe-off only hip extension angle increased during these strides and no significant changes occurred for the ankle and knee joints (Figure 3). The trunk–pelvis values decreased significantly across strides until stride nine. The maximal hip, knee flexion and dorsiflexion during the flight phase showed a similar development over the strides as the angles at touchdown (Figure 4). During the contact phase, only a few significant decreases in knee flexion and dorsiflexion over the different strides were observed.

**Figure 5:**
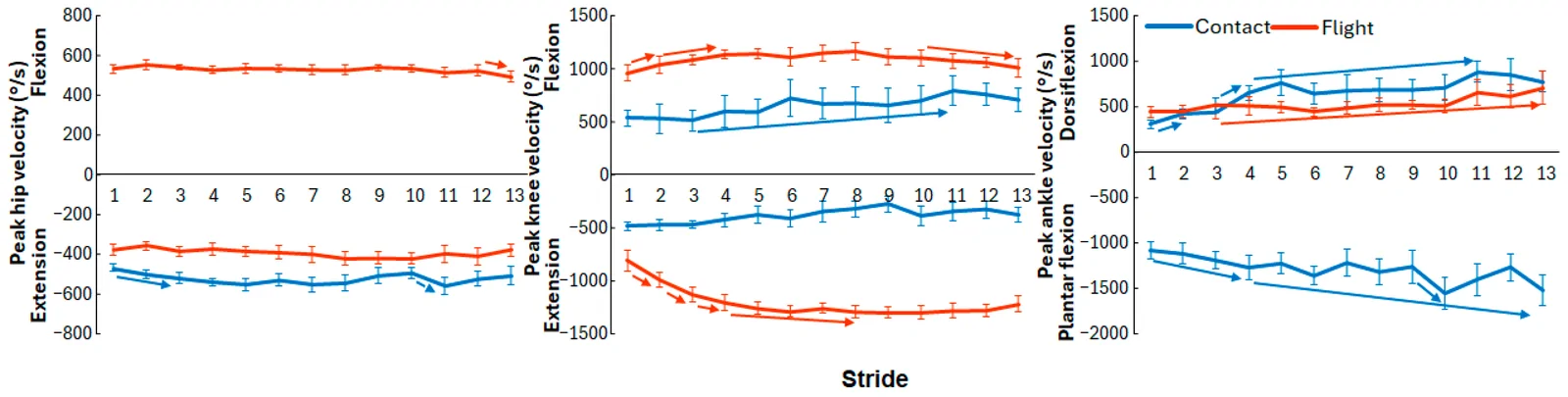
Mean ± 95% CI peak angular joint velocity during the contact and flight phase for the hip, knee and ankle for each stride. → indicates a significant change between these two strides and all subsequent data points located to the right of the arrow.

Also, all the peak angular joint velocities during the flight and contact phases were significantly affected by the strides with a large effect size (F ≥ 2.12, *p* < 0.002, η^2^ ≥ 0.14) (Figure 5). However, post hoc comparisons with Holm–Bonferroni correction revealed only significant increases in maximal dorsiflexion and plantar flexion velocities during the contact phase and dorsiflexion during the flight phase from strides 1–3 and again with the last stride. The maximal knee flexion and extension during the flight phase increased in the first 3–4 strides, then stabilized and knee flexion velocity decreased again between stride 10 and 13. Maximal knee flexion during contact time only increased significantly from stride 3 to stride 11, while peak hip flexion during the flight phase decreased significantly only at the final stride. Peak hip extension velocity decreased from stride 1 to 3 and then again from stride 10 to 11 (Figure 5).

### 3.3. Kinetics

All kinetic variables were affected by the strides (F ≥ 21.2, *p* < 0.001, η^2^ ≥ 0.59). Post hoc comparison revealed that anterior–posterior net force decreased significantly, while braking impulse increased with each stride. Propulsion impulse also decreased significantly during the initial strides but not the later strides of the 50 m, whereas vertical impulse decreased only between stride 1 and 2 and increased significantly again between stride 11 and 13.

**Figure 6:**
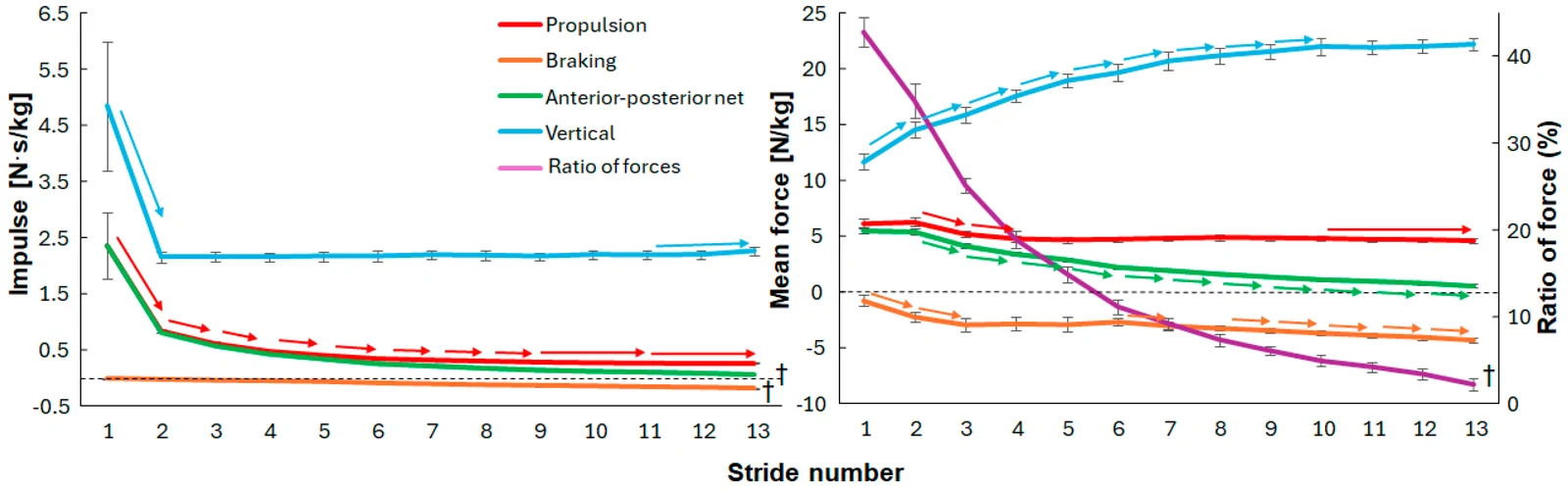
Mean ± 95 CI impulses, forces and rate of forces per kg body mass in different directions for each stride during the 50 m sprint. † Indicates a significant difference between each stride for this parameter. → indicates a significant change between these two strides and all subsequent data points located to the right of the arrow.

The mean vertical force increased each stride until stride 10, while the mean anterior–posterior net force decreased significantly each stride from stride 2 onwards. Furthermore, propulsion force decreased from stride 2 but significantly only until stride 4 and then again between stride 10 and 13. The mean braking force showed the opposite and increased significantly from stride 1 to 3, 6 to 7 and 8 to 13 each stride. This resulted in a significant decrease in ratio between the vertical and horizontal mean forces for each stride (Figure 6).

### 3.4. Electromyography

**Figure 7:**
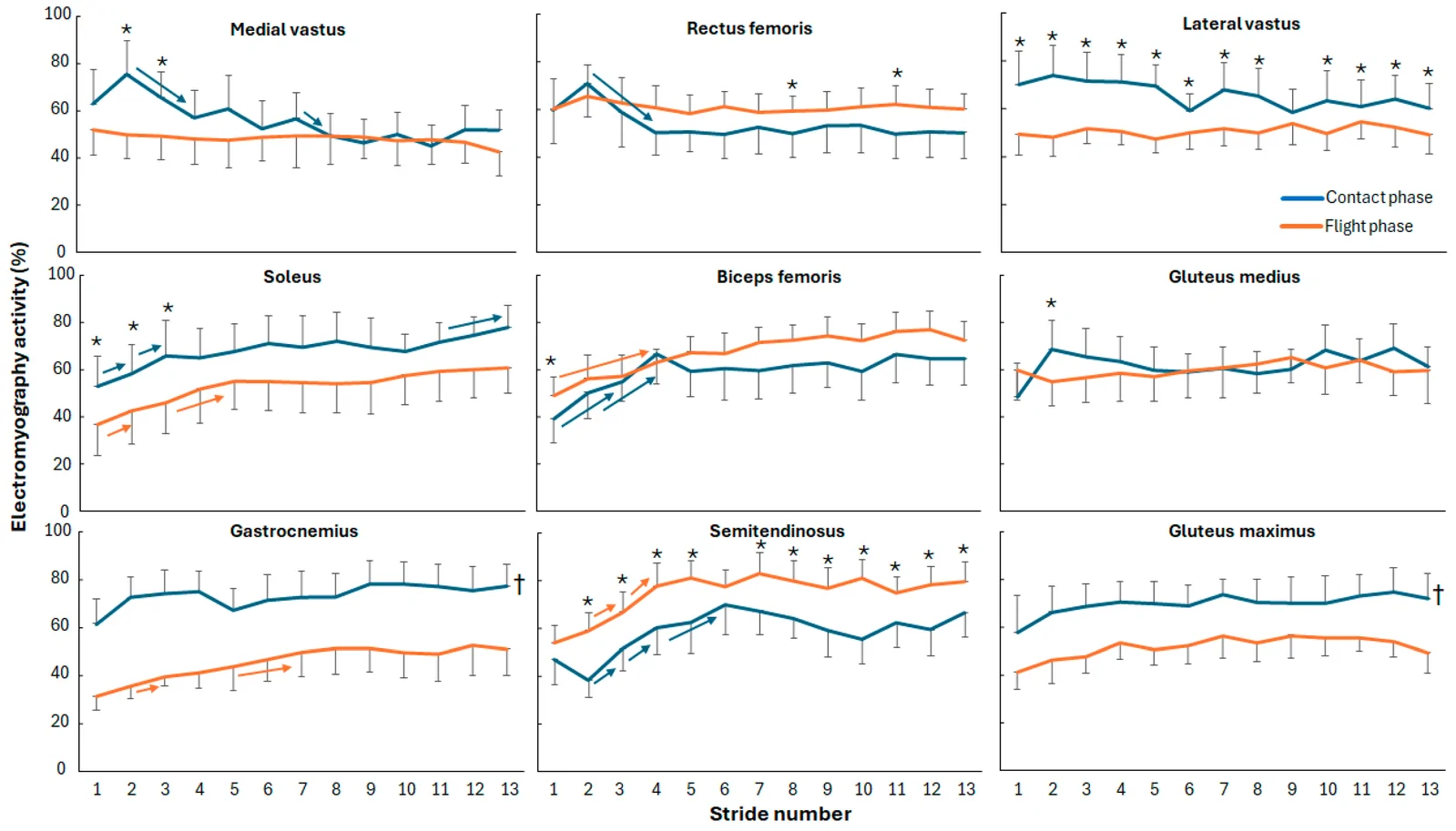
Mean ± 95% CI normalized RMS EMG activity for all nine muscles per stride specified in the contact and flight phase. * indicates a significant difference between the two phases for this stride. † Indicates a significant difference between the two phases for each stride for this muscle. → indicates a significant change in EMG activity between these two strides for this phase and all subsequent data points located to the right of the arrow.

A significant effect of strides was found for almost all muscles (F ≥ 2.66, *p* < 0.003, η^2^ ≥ 0.23) except for the vastus lateralis and both gluteus muscles (F ≤ 1.53, *p* < 0.118, η^2^ ≤ 0.16). There was also a significant effect of phase for the gastrocnemius, lateral vastus, semitendinosus and gluteus maximus muscles (F ≥ 16.22, *p* < 0.004, η^2^ ≥ 0.67), while a significant interaction effect was found only for both vastii muscles (F ≥ 1.87, *p* < 0.039, η^2^ ≥ 0.16). Post hoc comparison revealed that gastrocnemius and gluteus maximus EMG activity during the contact phase was significantly higher than that during the flight phase for every stride, while this was also observed in almost every stride for the lateral vastus. The EMG activity of the semitendinosus showed the opposite: higher activity during the flight phase compared with the contact phase in almost every stride (Figure 7). EMG activity increased significantly in both phases for the soleus muscle, especially between the first few strides, while for the gastrocnemius, only during the flight phase of the first few strides did EMG activity significantly increase. The three quadriceps decreased their EMG activity over the strides significantly (especially in the first few strides for the rectus femoris and medial vasti) during the contact phase but not during the flight phase, thereby causing a significant interaction effect for the two vastii muscles (Figure 7). Both hamstring muscles increased their EMG activity during the contact and flight phases, but mainly significantly over the first four strides, after which it stabilized (Figure 7).

## 4. Discussion

The aim of this study was to investigate the stride-by-stride development of spatiotemporal parameters, joint kinematics, kinetics, and EMG activity during a 50 m sprint in experienced sprinters. The main findings were that sprint acceleration was characterized by rapid early increases in stride frequency, joint angular velocities, and muscle activation, followed by progressive increases in stride length and reductions in contact time, leading to a plateau in velocity around 35 m. As speed increased, mechanics shifted from horizontal propulsion toward vertical force production, accompanied by stabilized kinematics and more efficient, coordinated neuromuscular activity.

Based upon the spatiotemporal changes during the 50 m sprint, it is possible to divide the sprint into three phases, as previously suggested by von Lieres und Wilkau, et al. [9], and in line with recent frameworks emphasizing phase-specific mechanical demands during sprinting [18]. The first phase is the initial acceleration, which extends from the start to around five strides in the current study (Figure 2). In this phase, the athlete accelerates to around 85% of maximal velocity (~12 m distance), which is mainly due to the combination of an increase in stride length and frequency (Figure 2). During this phase, contact time decreased with every stride, together with increased flight time. During the first 1–2 strides the propulsion impulse and mean forces were the highest and then decreased, while braking forces increased during these strides (Figure 6), which is in accordance with Morin, et al. [25]. This large propulsion impulse and forces could be caused by the high degree of forward leaning with the trunk, hips and knee and dorsiflexion at touchdown at low running speed. These lower-limb angles rapidly decreased each stride at touchdown (Figure 3). The increase in stride velocity was mainly caused by the increase in peak angular velocity in ankle joint and hip extension during the contact phase and faster repositioning of knee flexion and extension during the flight phase during the first few strides (Figure 5). This resulted in increases in hip extension angles during toe-off (Figure 3) during this phase of initial acceleration (Figure 5). To accomplish these fast joint movements, the medial vasti and rectus femoris are most active during the first stride and then decrease their activity during the landing phase, while the soleus during contact and the hamstring muscles (semitendinosus and biceps femoris) increase activity from stride to stride in this initial acceleration phase (Figure 7). This pattern aligns with previous EMG studies showing high early activation of the quadriceps muscles to stabilize the knee and generate propulsive force during ground contact, followed by a progressive shift toward greater reliance on the plantar flexors and hamstrings as velocity increases [14,16]. This transition likely reflects a redistribution of muscular contribution to optimize force production and limb repositioning as contact times shorten.

The second phase, referred to as the transition phase [9] is characterized by a further increase in stride velocity due to longer stride length and shorter contact times without any change in stride frequency (Figure 2). During this phase, the athlete adopts a more upright position in which the trunk and pelvis change with every stride, while the hip and knee angles slowly decrease over several strides during touchdown and the hip extension angle at toe-off slowly increases (Figure 3). During this phase, ankle joint angles do not change, but the increase in stride velocity seems to be caused by the slow increase in peak angular velocity of dorsal and plantar flexion during the contact phase over several strides and faster knee extension during the flight phase (Figure 5). These faster movements, together with the rising of the upper body over these strides, resulted in higher vertical forces, a decrease in propulsion, and lower anterior–posterior net impulse and forces (Figure 6). Thereby, the ratio of forces clearly decreases, reflecting a progressive shift toward more vertically oriented force production, which is consistent with the mechanical demands described in phase-specific sprint models [18,32]. In this context, leg stiffness becomes increasingly important, as Martín-Fuentes and van den Tillaar [30] showed that higher plantar flexion velocity at touchdown and toe-off is associated with faster sprint performance during this phase.

Muscle activation of the different muscles did not change much during the transition phase; only an increase in gastrocnemius activity during the flight phase was observed, which may indicate pre-activation prior to touchdown of the ankle joint to increase leg stiffness [33]. Since muscle activity changed very little during this phase, it may indicate that neuromuscular activation was already close to maximal. The higher semitendinosus activity during the flight phase is also well supported, as hamstrings are crucial for decelerating the swing leg and preparing for ground contact, as shown in several previous studies [34,35]. The lateral vasti, gastrocnemius and gluteus maximus muscles are clearly most active during the contact phase, as these muscles are mainly responsible for hip and knee extension and plantar ankle flexion (Figure 7) to propel the body forwards [36].

The last phase (from approximately 35 m onwards) during the 50 m sprint is the maximal-velocity phase [9], in which velocity is stabilized, while it seems that stride length still increases together with flight time. No changes in the lower-limb and pelvis angles seem to occur, while the trunk still seems to move more upright (Figure 3). Propulsion forces seem to decrease, while braking forces increase, resulting in a decrease in the anterior–posterior net force. Since the mean vertical force did not increase but the vertical impulse increased in this phase (Figure 6), this resulted in longer flight times. This increase in vertical impulse is probably caused by the increase in soleus activity during the contact phase as it is the only muscle of all the muscles that increases in activity during this phase. Also, during this phase, the maximal knee flexion velocity during the flight phase decreases. These two observations could indicate a compensation strategy for the body. As knee flexion velocity decreases, it could take a longer time to reposition the knee during the flight phase, so to compensate for this more vertical impulse is generated by the soleus muscle, thereby increasing flight time to keep the ideal posture of the knee for maximal velocity.

The present study has some limitations. Only a small sample consisting of male athletes was tested from a specific performance level. Therefore, the findings may differ for sprinters of other performance levels or a different sex, especially for elite sprinters as they in general accelerate longer and have possibly higher kinetic and kinematic variables resulting in shorter sprint times. Consequently, the findings of the present study cannot yet be generalized. Furthermore, there was a lot of variability in EMG measurements between the different athletes, which makes it difficult to compare all of the measurements with each other. As normalization of EMG activity was done by using the highest mean RMS of a phase for each muscle, it was impossible to compare normalized muscle activity between the different muscles. In addition, only 50 m sprints were performed, in which three phases were identified. However, longer sprint distances also involve a deceleration phase [37]. In these longer distances it would be interesting to investigate these parameters to get more insight about potential compensatory strategies. Therefore, future studies should investigate sprints over longer distances and investigate longitudinal changes in sprint performance in relation to changes in kinematics, kinetics and EMG activity to gain more insight into the possible mechanisms behind it for each athlete.

## 5. Conclusions

By integrating spatiotemporal, kinematic, kinetic, and EMG data, this study extends the existing literature by providing a detailed stride-by-stride perspective on how these variables interact during 50 m sprints. The findings suggest that 50 m sprints can be described by three phases (initial acceleration, transition, and maximal velocity) as a descriptive interpretation of the observed stride-by-stride patterns, supported by the previous literature, and involving tightly coordinated changes across multiple biomechanical domains. Initial acceleration (first 3–5 strides) is dominated by rapid adjustments of increased stride frequency, rapid changes in joint angles and rising EMG activity in key muscles. These results reflect a neuromuscular strategy focused on maximizing horizontal force production and rapid limb repositioning. The transition phase (up to ~30–35 m) continued progressive increases in stride length and was further characterized by progressive decreases in contact time with enhanced leg stiffness, progressive decreases in horizontal force contribution and stabilization of kinematics and EMG. In the maximal-velocity phase there was a plateau in velocity, increased flight time, greater vertical force reliance (increased vertical force relative to horizontal force), and neuromuscular activation patterns became more stable. This phased progression supports contemporary biomechanical models of sprinting and emphasizes the importance of both mechanical and neuromuscular efficiency in achieving optimal sprint performance. Collectively, the findings suggest that sprint performance is determined not by isolated variables but by the dynamic coordination of mechanical and neuromuscular systems, which evolve in a phase-specific manner across successive strides.

From a performance perspective, these findings reinforce the importance of phase-specific training strategies. Initial acceleration training should emphasize rapid force production and stride frequency, while training for the transition phase should focus on stride length development and efficient force application.

## Data Availability

The data presented in this study are available on request from the corresponding author. The data are not publicly available due to national laws of the Norwegian government on privacy.
